# Making governance work in the health care sector: evidence from a ‘natural experiment’ in Italy

**DOI:** 10.1017/S1744133115000067

**Published:** 2015-03-30

**Authors:** Sabina Nuti, Federico Vola, Anna Bonini, Milena Vainieri

**Affiliations:** Laboratorio Management e Sanità, Institute of Management, Scuola Superiore Sant’Anna, Pisa, Italy

## Abstract

The Italian Health care System provides universal coverage for comprehensive health services and is mainly financed through general taxation. Since the early 1990s, a strong decentralization policy has been adopted in Italy and the state has gradually ceded its jurisdiction to regional governments, of which there are twenty. These regions now have political, administrative, fiscal and organizational responsibility for the provision of health care. This paper examines the different governance models that the regions have adopted and investigates the performance evaluation systems (PESs) associated with them, focusing on the experience of a network of ten regional governments that share the same PES. The article draws on the wide range of governance models and PESs in order to design a natural experiment. Through an analysis of 14 indicators measured in 2007 and in 2012 for all the regions, the study examines how different performance evaluation models are associated with different health care performances and whether the network-shared PES has made any difference to the results achieved by the regions involved. The initial results support the idea that systematic benchmarking and public disclosure of data are powerful tools to guarantee the balanced and sustained improvement of the health care systems, but only if they are integrated with the regional governance mechanisms.

## 1 Introduction

Following the wave of the international New Public Management movement (Hood, 1991), many health care systems underwent reforms in the 1990s to shift control from the national to the local level, and thus increase the scope for flexibility in local governance (Fattore, 1999; Saltman *et al.*, 2007). Since then, reforms based on New Public Management principles have been aimed at making the public sector more efficient, effective and accountable (Hood, 1995; Lapsley, 1999; Saltman *et al.*, 2007). Some countries have introduced quasi-market mechanisms to foster competition. Others have focused on measuring performance, which has become a mantra at all levels of government since the 1990s (Radin, 2000). As a consequence, health systems and institutions have adopted different strategies and governance models with a particular interest in measurement tools and techniques. Initially, performance measurement focused on financial issues and neglected multiple strategic objectives to drive change (Ghobadian and Ashworth, 1994; Pollitt and Bouckaert 1995; Guthrie and English, 1997; Lorden *et al.*, 2008). Thus, comprehensive multi-dimensional performance measurement frameworks, such as the balanced scorecard, were introduced (Kloot and Martin, 2000; Yang and Tung, 2006). Another development was the benchmarking of health performance measurement systems at international, national and local levels (NHS Executive, 1999; Pink *et al.*, 2001; Johnston, 2004; Vainieri and Nuti, 2011). Benchmarking can help managers learn from best practices (McNair and Leibfried, 1992) and be used as a mechanism to detect unwarranted variations and encourage their reduction (Arah *et al.*, 2003).

We argue here that to drive health care system improvements at national or local levels, the performance evaluation system should be aligned with the national (or sub-national for local governments) strategy, mission and vision in order to provide coherent messages for those running the units and their employees (as suggested by Ferreira and Otley, 2009).

Relying on previous studies (Cromwell *et al.*, 2011; Brown *et al.*, 2012; Bevan and Fasolo, 2013; Bevan and Wilson, 2013), we identify five governance models:

1. The ‘trust and altruism’ model relies on the perspective that all public servants behave like knights. This was the traditional model applied by the National Healthcare System (NHS) and does not focus on success and failure. On the contrary, it may reward failure and ignore success.
2. The ‘choice and competition’ model is based on the quasi-market system where patients can choose and the money follows the patients. This model introduces external incentives, and patients (or insurance companies) can choose providers on the basis of quality information.
3. The ‘hierarchy and targets’ model, also known as ‘command and control’, is based on recourse to external incentives and the strong role of performance management (generally by the central government). It has side effects such as high monitoring costs and low acceptance by professionals.
4. The ‘transparent public ranking’ model is based on the lever of reputation. This model has been applied in England, where it is known as the ‘naming and shaming’ model.
5. The ‘pay for performance’ (P4P) model draws upon economic incentives to direct the managers’ behaviour. Regarding the specific use of the expression ‘pay for performance’ in this paper, the Italian regional governments that adopt the ‘P4P’ model link the rewarding scheme of their health authorities’ (HAs) CEOs to the performance they achieve. This model is based on the assumption that financial payments can motivate people to achieve performance targets. It aims to improve quality and efficiency by paying more for results or actions such as evidence-based preventive care services, or denying payment for preventable complications.

These governance models can be adopted at the macro level by the state, at the meso level by regions (or counties and provinces, depending on a country’s organization) and at the micro level by local institutions (municipalities, health care authorities, hospitals, etc.). The basic ingredients of the five ‘ideal typical’ models can be mixed. For instance, Bevan and Fasolo (2013) have described the ‘star rating’ model, which was applied by the English NHS from 2000 to 2005, as a combination of the third and fourth models.

This paper discusses which governance models have been adopted by the Italian regions (the meso level) and their impact on performance. The paper first classifies the regions using the five governance models described above. Second, it describes the Inter-Regional Performance Evaluation System (IRPES), which is an evaluation tool currently adopted by a network of 10 Italian regions and how this was used in terms of a governance model between 2006 and 2012. Third, this paper examines how the performance of the regions changed between 2007 and 2012. The paper concludes by discussing the outcomes of the different governance models adopted by the regions and, in particular, how IRPES has, or has not, driven improvement in the network.

## 2 The governance systems adopted by Italian regions in the health care sector

The Italian NHS, which follows the Beveridge model, is a public health system that provides universal coverage for comprehensive and essential health services through general taxation. Since the early 1990s, a strong decentralization policy has been adopted in Italy and the state has gradually ceded its jurisdiction to its 20 regions (France and Taroni, 2005).^1^

The central level – represented by both the Ministry of Health and the Ministry of Finance – ensures that the regions keep their health care expenditure within their budgets and guarantees the essential levels of care. Since the 2000s, the health care budget has been allocated to the regions on the basis of a per capita share, partially adjusted by the age distribution of the population. On the other hand, the regions are in charge of organizing health care services. They define their own regional health plans, coordinate the strategies of the regional HAs, and allocate the budget within their systems. Since 2000, the regions have become more fiscally autonomous and more financially responsible (Ferrario and Zanardi, 2011; Ferrè *et al.*, 2012).^2^

Italian regions now have the political, administrative and financial responsibility for the provision of health care to their residents.

De Vries (2000) argued that the results of decentralization depend on the cultural and political context, on the administrative capabilities of the actors involved and on how the process is promoted (see also Putnam, 1993). The consequence is that there are now 20 regional health care systems (RHSs) in Italy with different governance models and management tools (Formez, 2007; Censis, 2008; Tediosi *et al.*, 2009; Vainieri and Nuti, 2011; Carinci *et al.*, 2012; Mapelli, 2012). France *et al.* (2005) highlighted the north–south performance disparities in mortality, expenditure and equity up until 2002. About 10 years later, Toth (2014) reviewed the first decade of Italian decentralization (1999–2009) and concluded that the shift of power from the central to the regional level had accentuated the north-south divide, in terms of expenditure and perceived quality of health care services.

The high degree of geographical variation in various measures of performance demonstrates that these general conditions and quality/volume standards are not equally achieved among the Italian regions. Such variation is common in health care systems (Wennberg and Gittelsohn, 1973; Wennberg, 1999; Wennberg *et al.*, 2002; Appleby *et al.*, 2011; Corallo *et al.*, 2014; EuroHOPE, 2014; OECD, 2014).

During the first years of devolution (2001–2005), the central government bailed out the previous health care deficits of the regions. In order to prevent increasing deficits, the Italian government approved legislation that introduced a new recovery process to reduce the financial deficit of the RHSs (Bordignon and Turati, 2009; Ferrè *et al.*, 2012). The Financial Stability Law L. 311 (30 December 2004) and the Financial Stability Law L. 296 (27 December 2006) regulated the design and the adoption of the recovery plans.^3^

The laws decree that if the regions are in deficit – even with extra finance from regional taxes – they have the right to access a bail out fund, financed by national taxation. To access this fund, the regions are required by the central government to produce a recovery plan, which should identify strategic actions to address the structural determinants of the costs involved in achieving financial balance (Ferrè *et al.*, 2012). These recovery plans are subject to approval by the National Ministry of Health and by the Ministry of Economy and Finance. If the plans are deemed inadequate, the President of the region is formally replaced by an ‘*ad acta* commissioner’, that the law states to be the president himself, and regional taxes have to be automatically increased up to a predefined threshold. Since 2007, 10 out of 20 RHSs have carried out a recovery plan: Abruzzo, Molise, Apulia (since 2010), Campania, Calabria (since 2009), Sicily, Lazio, Piedmont (since 2010), Sardinia and Liguria. In five of these RHSs, the central government has nominated a commissioner in charge of local implementation. So far, Liguria and Sardinia have successfully implemented their recovery plans with a balanced budget (these regions have succeeded by reallocating financial resources from other public sectors to health care).

We now describe the models of governance adopted by each region, illustrating how the regions combined the five above-mentioned ‘ideal typical’ models from 2007 to 2012. We identified four groups of regions, according to how they mixed the five governance models.

First, Lombardy is the only region that opted for the ‘choice and competition’ model by splitting purchasers and providers (including private institutions) in order to stress the role of patient choice to boost competition (Lombardy Region, 1997). The principal tools adopted by Lombardy to manage its services are represented by (a) tariffs; (b) the adoption of the Joint Commission International Accreditation Program for Hospital Care (JCI, 2014); (c) hospital care outcomes and patient satisfaction (Vittadini, 2012). General managers of the LHAs are rewarded according to the achievement of targets negotiated with the regional administration.^4^ Lombardy, therefore, combines some elements of the ‘choice and competition’ model (tariffs and patient choice) with ‘pay for performance’. However, despite the link between CEO rewards and performance results, the variability in managers’ results and the related economic incentives is low, thus weakening the P4P strategy as a governance tool (Vainieri *et al.*, 2013). Finally, although Lombardy measures outcomes in benchmarks, it does not fully disclose the results either to the hospitals, or to the patients. Lombardy hospitals are the only ones to know their own results, without knowing how they compare with each other. They therefore cannot identify and learn from the best practices (Vittadini, 2011; Berta *et al.*, 2013). Moreover, despite the alleged stress on patient choice, Lombardy does not use transparent public ranking and does not publicly disclose results.

Second, the ‘hierarchy and targets’ (or ‘command and control’) model has been applied by the state for the following eight regions that are still subject to recovery plans: Abruzzo, Molise, Apulia, Campania, Calabria, Sicily, Lazio and Piedmont. Although the central government specifies financial targets for all of them, the response differs according to the previous governance models and the managerial skills of the staff. Irrespectively of these differences, none of these eight regions nor the state, systematically benchmark the clinical results between regions, nor do they publicly disclose data.

Recent studies in Italy on top management evaluation systems have highlighted that the setting of targets phase in these regions does not follow objective and rational processes (Caldarelli *et al.*, 2013; Vainieri *et al.*, 2013). Indeed, they often do not take into consideration past performance and largely depend on qualitative targets, which are usually vague and can be interpreted in different ways (Vainieri *et al.*, 2013). Moreover, these regions lack a reliable supervision and monitoring system (Ferrè *et al.*, 2012). Sanctions for failure are therefore not clearly applied and the ‘hierarchy and targets’ (or ‘command and control’) model appears to be applied loosely. Thus, the difference with the ‘trust and altruism’ model (which is not formally adopted by any Italian region) is blurred.

Third, since 2006 Tuscany and an increasing number of regions (10 regions in 2014) have adopted a mixed governance model that combines ‘hierarchy and targets’ with ‘transparent public ranking’ (in the form of public disclosure of performance data) and ‘pay for performance’ (limited to the CEOs’ rewarding schemes). This mixed model has been adopted at different times between 2007 and 2014 by Tuscany, Liguria, Umbria, Basilicata, Trento, Bolzano, Marche, Veneto, Emilia Romagna and Friuli.

In addition, another two regions – the Aosta Valley and Piedmont – joined the Tuscan Performance Evaluation System for three years, from 2008 to 2010. As previously mentioned, Piedmont partially lost its autonomy when it shifted to the command and control model, with the strong role of central government, while the Aosta Valley went back to its regional model of hierarchy and targets, mainly focused on epidemiological issues. The Aosta Valley has disclosed performance results to internal users rather than to the public. These results are not always translated into policy decisions (Carinci *et al.*, 2012).

Fourth, Emilia Romagna, Veneto and Friuli have only recently opted for the governance model suggested by Tuscany. Before 2014, they had applied mixed governance models in different ways (Vainieri and Nuti, 2011; Carinci *et al.*, 2012). Between 2007 and 2012, all three regions used some performance evaluation mechanisms regarding several dimensions. For instance, Friuli linked health databases, delivering detailed reports and regular publications for internal users. Veneto conducted patient surveys, and Emilia Romagna conducted self-evaluation cycles, involving health professionals (Vainieri and Nuti, 2011; Carinci *et al.*, 2012). However, these tools were not systematically used in regional decision making and their performance was not benchmarked against the other regions’ performance.

In conclusion, no regional government in Italy can be considered to have adopted one single clear-cut governance model, but rather a combination of them.

In this context, the experience of the regions that have adopted the same Performance Evaluation System is worth examining.

### 2.1 The network experience

**Figure 1:**
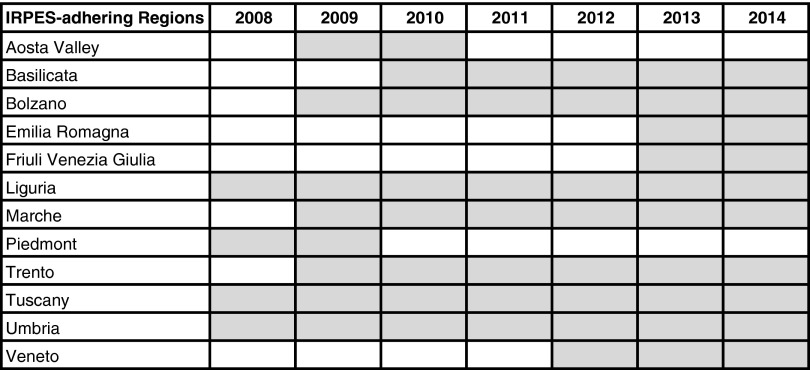
Regional adhesion to Inter-Regional Performance Evaluation System (IRPES).

Since 2008, a growing number of regions have adopted the same IRPES, which was designed and implemented for the first time in 2005 in all of Tuscany’s local health authorities (LHAs) by the Laboratorio Management e Sanità (MeS) of the Scuola Superiore Sant’Anna to measure and monitor indicators of quality, efficiency, appropriateness, continuity of care, patient satisfaction and staff satisfaction (Nuti and Bonini, 2011, 2013; Nuti *et al.*, 2013). In 2014, there were 10 regions in the network: Basilicata, Liguria, Marche, the Autonomous province of Bolzano, the Autonomous province of Trento, Toscana, Umbria, Veneto, Emilia Romagna and Friuli Venezia Giulia. The regions joined the network in different years, as reported in Figure 1.

The Laboratorio MeS develops the performance evaluation framework and brings objectivity to the benchmarking processes as an independent research unit. It coordinates and manages information sharing and data acquisition. The 10 regions in the network agree on the indicators for the benchmarking and on how they should be calculated. Each region is responsible for processing its own data, in order to increase the awareness and the expertise of the regional managers and their staff.

The aim of the IRPES is to assess and monitor health system performance at a regional and local level: the results are shown by region and by HAs (both LHAs and teaching hospitals). In 2014, IRPES monitored the performance of 99 HAs.

The regional network integrates a *longitudinal* (the trend) with a *cross-sectional* perspective, based on the benchmarking process. It provides the regions with valuable information in order to define priorities and fix appropriate targets, considering the results in benchmarking. In addition, given that they follow the same PES, the regions can evaluate, share and spread best practices.

Indicators are defined by endorsing a ‘managerial’ perspective aimed at organizational improvement (Mannion and Davies, 2008). The rationale behind the selection of each indicator is the informational contribution it can offer the managers and policy makers. Indicators are chosen not only because they represent the epidemiological situation of single regions/Local Authorities, but because they also detect best (organizational) practices or, on the contrary, flawed clinical processes.^5^

Indicators are defined in regular meetings with regional representatives that include both managers and clinicians. For an evaluation system to be able to influence and change behaviours, it must actually win support from clinicians on the rules and criteria their performance is measured against (Locke and Latham, 2013).

PES encompasses a large set of indicators that are up-to-date because they are calculated and disseminated in a six-month period. The indicators are grouped into 60 indexes and classified in six dimensions (a letter is used to indicate each dimension):

A. *Population health*.
B. *Regional strategy compliance*, to guarantee that strategic regional goals are pursued in the time and manner indicated.
C. *Quality*, appropriateness, continuity of care, patient safety and managing supply to match demand.
D. *Patient satisfaction*, the patients’ experience and level of satisfaction with health services.
E. *Staff satisfaction*, results of surveys on the satisfaction level of staff with their working conditions and management.
F. *Efficiency and financial performance*.

**Figure 2:**
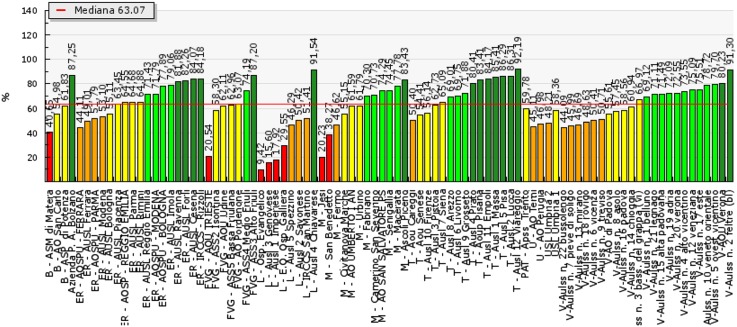
Percentage of femur fractures operated on within two days.

PES measures results in quantitative terms and then assesses performance for 100 of the 160 indicators: excellent, good, sufficient, poor or very poor. These five evaluation tiers are associated with different colours, from dark green (excellent performance), to red (poor). Regions use the same reference standards for evaluation, based on the scientific literature, national standards or, where these are lacking, on the median of the 99 HAs. Figure 2, as an example, displays the indicator of femur fractures operated on within two days.

**Figure 3:**
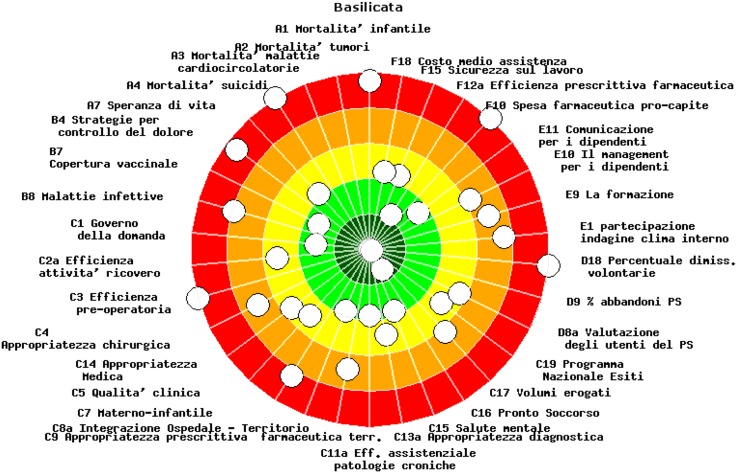
The ‘dartboard’.

In order to show the performance of each region or HA, a chart with the six dimensions is used (see Figure 3). The chart is also divided into five evaluation bands, associated with different scores and colours as explained above. Each indicator is positioned on the chart and there is no overall unique ranking for regions/HAs. When the result has a high score, it is displayed close to the centre (dark green), and when the score is low, it is displayed far from the centre (red).

The number of indicators varies by region, because each region chooses which ones to include, with reference to local context and strategies. However, there is a core group of indicators that all the regions consider mandatory for the main pillars of the health care system. Indeed, the majority of indicators are common to all the regions because the main objectives are the same at the national level. The IRPES structure also allows regions to choose different indicators to reflect the different regional strategies. The inclusion of a specific indicator within IRPES signals the strategic relevance the indicator is deemed to have, for all the regions or for a subset of them.

From the beginning, the regional network agreed on transparency for public accountability. An annual performance report is published and the web platform where data are stored is public (http://performance.sssup.it/network). The report includes all the regions, and local performance (HAs) is also shown.

There are regular meetings between the regional representatives to share the results of the assessment system, identify best practices and compare outcomes of different regional strategies. The systematic reporting of comparisons of performance that IRPES provides, may result in some element of competition among the regions. Working groups are established as issues arise to discuss the different impacts of policies and to develop new indicators.

## 3 IRPES as a governance tool

Several governance models can exploit IRPES data (Brown *et al.*, 2012; Nuti *et al.*, 2013). With reference to the five above-mentioned ‘ideal typical’ models:

1. IRPESs can be linked to strategic planning and HAs’ goal setting so that it is integral to political accountability. The IRPES provides a basis for regions to identify priorities and to set challenging targets. It can therefore be used as a tool to sanction managers according to their performance (‘hierarchy and target’ governance model).
2. IRPESs can be linked to the CEOs’ financial reward system. Indeed, it is largely acknowledged that reward schemes reinforce orientation and directions. Hence, performance indicators monitored and assessed by IRPES can be included in CEO schemes in order to better align CEO objectives with those of the institution and of the health care system in general (‘pay for performance’ governance model).
3. Regions can use IRPES information as an improvement tool to leverage their reputation, by publicly disclosing data to all the stakeholders within the regional health system (‘transparent public ranking’ governance model). Regions can disseminate results through public events, such as press conferences, meetings and internal periodic monitoring. To enable peer review mechanisms, the performance results can be discussed in all kinds of contexts such as managerial training activities for top and middle management, in order to stimulate feedback from professionals who are the basic operators of change.
4. IRPES can be used as a tool to align the three above-mentioned governance mechanisms (mainly addressed to managers) to the operative units of the RHSs. IRPES results can also be integrated within the budgeting process of HAs.

The integration and the joint adoption of all these strategies provide a boost to improve performance, as demonstrated by the comparison of Lazio and Tuscany regarding hip fractures operated on within two days (Pinnarelli *et al.*, 2012).

We now describe how IRPES-adhering regions have integrated the PES with their internal governance mechanisms in different ways.

Tuscany and Basilicata are currently using all four strategies, and Veneto, Emilia Romagna and Friuli seem to be on the same track. Piedmont has also applied all four strategies but only when it participated in the network.

Trento, Liguria and Umbria, have adopted three of the four strategies. Trento has linked IRPES to other governance tools (strategy 1), CEO reward schemes (strategy 2) and both internal and public events (strategy 3). Full integration in the local budget process is still lacking. Liguria has also applied the first three strategies but in a non-systematic way. Finally, Umbria has adopted the first two strategies and partially introduced IRPES in its managerial training programmes.

Finally, Bolzano, Marche and the Aosta Valley have not endorsed any of the aforementioned four strategies. Table 1 summarizes the different governance models adopted by the regions in 2007–2012.

**Table 1:** The regional governance models between 2007 and 2012

	Trust and Altruism	Choice and Competition	Hierarchy	Pay for performance	Transparent public ranking
Abruzzo	✓		✓		
Aosta Valley	✓		✓		
Apulia	✓		✓		
Autonomous Province of Bolzano	✓				
Autonomous Province of Trento			✓	✓	✓
Basilicata			✓	✓	✓
Calabria	✓		✓		
Campania	✓		✓		
Emilia-Romagna			✓	✓	
Friuli-Venezia Giulia			✓	✓	
Lazio	✓		✓		✓
Liguria	✓		✓		✓
Lombardy		✓		✓	
Marche	✓				
Molise	✓		✓		
Piedmont	✓		✓	✓	
Sardinia	✓		✓		
Sicily	✓		✓		
Tuscany			✓	✓	✓
Umbria	✓		✓	✓	
Veneto			✓	✓	

## 4 Methodology

To compare the results achieved by the regions with different governance models, we chose 14 performance indicators measured in 2007 and in 2012 (see Table 2).

**Table 2:** Set of selected indicators

Indicator code	Indicator label
	*Hospital care (H)*
H1	Ordinary hospitalization rate
H3	Percentage of medical DRG from surgical departments
H4	Percentage of laparoscopic cholecystectomies in day surgery or 0–1-day admissions
H5	Surgical essential levels of health services DRG – standard percentage achieved
H9	Percentage of caesarean births
H11	Percentage of femur fractures operated within 2 days
H13	Preoperative average hospital stay
H14	Percentage of short medical hospitalizations
	*Primary care (T)*
T2	Hospitalization rate for heart failure (50–74 years old)
T3	Hospitalization rate for diabetes (20–74 years old)
T4	Hospitalization rate for COPD (50–74 years old)
AF5	Per capita net pharmaceutical expenditure
	*Preventive care (P)*
P3	Mammography screening extension
P4	Compliance with mammography screening

These specific indicators were chosen because they had already been validated by the pilot study coordinated by the MeS Laboratory in 2009, on behalf of the Italian Ministry of Health (Nuti *et al.*, 2012). Most of the indicators were derived from the framework already developed by the Tuscany region or from international studies (Canadian Institute for Health Information, 2001; OECD, 2003; WHO, 2003; AHRQ, 2006; Department of Health, 2008) and they were selected on the basis of the following criteria (Kelley and Hurst, 2006):

∙ relevance in terms of policy;
∙ scientific soundness of the indicators in terms of their validity and reliability;
∙ feasibility of obtaining nationally comparable data;
∙ ability to provide a comprehensive overview of hospital, primary and preventive care in the Italian health care system.

The national hospital discharge database for the years 2007 and 2012 was used for all the measurements on hospital and primary care dimensions. The 2007 and 2012 OsMed reports were used for the indicators on pharmaceutical care (OsMed, 2007; OsMed, 2012). The 2007 and 2012 national screening reports were used for the measurements on prevention (National Screening Observatory, 2014). Preventable hospitalization rates for chronic conditions from inpatients data were used as a proxy of primary care performance because of the lack of national comparable sources on territorial services (Ricketts *et al.*, 2001). Indicators from hospital inpatient data, when possible, were standardized according to sex and age, using Italian residents in 2001 as a standard population.

The indicators refer to two years – 2007 and 2012 – and provide information for a pre–post comparison: IRPES was actually first developed in 2008.

All the selected indicators were considered by the IRPES regions to have the same importance in measuring the performance of the RHS. This set of indicators therefore offers a preliminary overview of the differences across regional health care performances and how they shifted in the 2007–2012 period.^6^

In order to summarize regional performances in 2007 and 2012, the 14 indicators were combined into a single indicator according to the following methodology:

∙ we ranked each indicator for each year (2007 and 2012);
∙ we assigned the quintile each region occupied for each specific indicator;
∙ coefficients ranging from 0.2 (worst performing), 0.4 (badly), 0.6 (average), 0.8 (well) to 1 (best) were then assigned. For each region, the weighted indicators were first summed and then divided by 14 (the total number of indicators), obtaining a performance score that hypothetically ranged from 0.2 (all the 14 indicators in the worst quintile) to 1 (all the 14 indicators in the best quintile). This procedure was applied both to the 2007 and 2012 indicators.

The overall performance score is the mean of the 14 (ranked and weighted) indicators. Although we limited ourselves to 14 indicators in devising the performance score, this was supported by the decision of all the IRPES regions to consider the indicators as equally important and relevant in terms of offering an overview of performance of the RHSs. In addition, according to the national legislative framework, the three health care levels – hospital, primary and preventive care – should be financed according to fixed shares (respectively: 44, 51 and 5%), which mirror their respective importance (State-regional Conference, 2009; Presidency of the Republic of Italy, 2011). The proportion of the selected indicators approximately reflects this balance (although slightly overestimating the importance of hospital care). Finally, note that the overall performance score is not conceived as a tool to rank the regions, but as an explanatory expedient used to offer an overview of their performance in the 2007–2012 period.

First, the method allows for cross-regional comparisons, regardless of the scale of each indicator, and offers an overview of the performances of the RHSs. Second, it allows for longitudinal comparisons (2012 vs 2007) that are not affected by different regional starting points in 2007 and by events at a national level, as trends are assessed in relative terms, in relation to all those of the regions.

**Figure 4:**
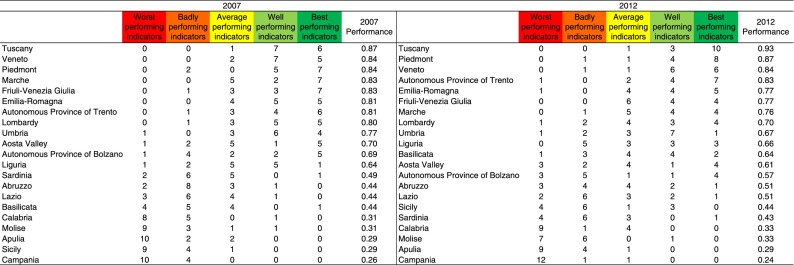
Regional performances in 2007 and in 2012.

**Figure 5:**
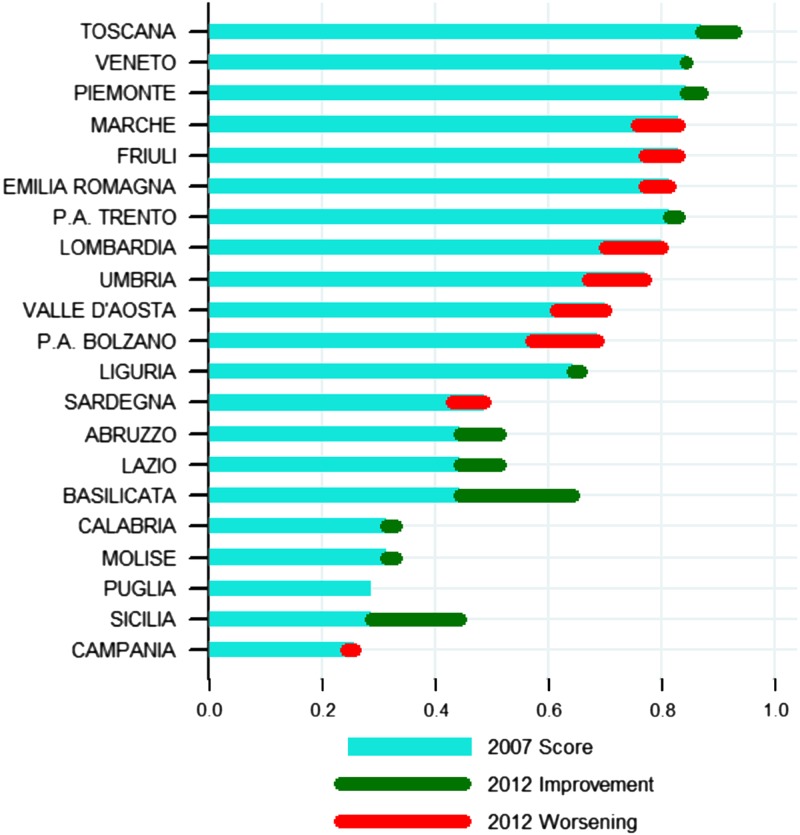
Regional performance.

Figures 4 and 5 show the regional performances in 2007 and in 2012. Figure 4 overviews each region’s performance in 2007 and 2012, by listing the number of indicators according to the quintile they occupied.

The dynamics of each region’s relative performance is shown by Figure 5. The blue line shows the 2007 score; the green and red lines portray each region’s improvement or lack of, respectively, between 2007 and 2012.

The above-mentioned methodology and its graphic representation offer some preliminary insights into impact of the different governance models adopted by the regions on performance and the relevance of the IRPES tool. The variability in the governance models adopted in accordance with the above-mentioned decentralization process, actually provides a natural experiment to study the association of regional health care performances with different governance models.

Other regional variables might be associated with health care performance and could be a confounding factor for our analysis. The Italian regions have historically been heterogeneous in terms of size, population, economic development, civic culture and institutional performance, with a very clear difference between the north and the south of the country (Putnam, 1993; Cotta and Verzichelli, 2007; Pavolini and Vicarelli, 2012; Toth, 2014). From an economic point of view, despite the substantial difference between the northern regions (with a per capita income of 27,500 euros in 2012) and the southern regions (18,200 euros), this disparity is not reflected in their public health spending (Istat, 2012). As mentioned above, since the 2000s, the health care budget has been allocated among the regions on the basis of a per capita share, partially adjusted by the age distribution of the population. Therefore, all regions are roughly guaranteed the same per capita resources for health care (Toth, 2014).

## 5 Discussion

In Section 2, we grouped the Italian regions into four clusters, according to how they mixed the five ‘ideal typical’ governance models previously outlined. We will now discuss the performance of each group in 2007–2012, according to the methodology explained in Section 4.

As already mentioned, Lombardy is the only region that adopted a ‘choice and competition’ governance model. According to the 14 indicators we considered, this governance system does not seem to be associated with outstanding performances in 2012, or with exceptional improvement. Lombardy actually performed slightly better in 2012 than the other regions but had actually got worse compared with 2007. Hospital-related performance seems to be detrimentally affected by this governance model, both regarding appropriateness (H3, H4, H5) and quality indicators (H9 and H11). Both the regions with a ‘trust and altruism’/‘hierarchy and targets’ governance model (debt-rescheduling plan) and those that chose to adhere to a ‘hierarchy and targets’/‘transparent public ranking’/‘pay for performance’ (IRPES) show different performances, suggesting that governance models can be applied differently and may be affected by other regional characteristics.

Regions with a recovery plan (group 2) generally show a poor performance in 2007 and some degree of improvement from 2007 to 2012. Hence, it seems that the strict commitment of the central government to setting targets and controlling their achievement has pushed regions towards improving their performance. However, there are doubts as to the real effectiveness of the regional recovery plans, which are more oriented towards financial expenditure and hospital performance rather than the quality of services. Sicily and Piedmont represent two interesting cases. Sicily registered one of the worst performances in 2007 but achieved a significant improvement from 2007 to 2012. However, the analysis of single indicators shows that improvements almost uniquely refer to hospital care indicators. Primary and preventive care, which were poor in 2007, did not significantly improve in 2012 and the same goes for pharmaceutical expenditure.

On the one hand, Sicily’s improvement may be due to the introduction of a clause in top managers’ contracts, which required the achievement of specific performance targets linked to the national outcome evaluation program (PNE) run by AGENAS (the National Agency for Regional Health Services). Target achievement is one of the conditions needed to have appointments confirmed: the commitment to strict ‘hierarchy and targets’ models therefore seems to be associated with a significant performance improvement. The Sicilian case suggests that ‘hierarchy and targets’ models prove to be more effective in dealing with hospital care re-organization, where structural reforms require strong political commitment, while it might be more difficult to deal with primary and preventive care.

On the other hand, these results seem to confirm the findings of Ferrè *et al.* on the above-mentioned evaluation of regional recovery plans (Ferrè *et al.*, 2012). Complex systems may entail the hierarchy model being integrated with different governance models (e.g. ‘transparent public ranking’ and ‘pay for performance’) that help align the different goals of the powerful players with regional goals.

Piedmont is a northern region that has generally shown high-quality performances. It adhered to the IRPES network in 2008 and left it in 2010, when it entered the debt-rescheduling plan. Despite the recovery plan, it seems that the Piedmont health care system was able to ensure increasing quality performances. Indeed, the region improved hospital performances (see indicators H4, H11, H13) confirming, at the same time, its excellent primary care. IRPES-adopting regions (group 3) showed different internal patterns. They were, in general, characterized by higher performances (both in 2007 and in 2012) than the regions with recovery plans, however, they showed significant variability, especially in their dynamics. The two regions that improved the most were Basilicata and Tuscany.

Regarding Basilicata, single indicators highlight more balanced dynamics than Sicily. There were improvements in the three assistance levels (hospital, primary and preventive care), although a couple of hospital-care indicators – referring to appropriateness – worsened (H3 and H5).

The second interesting case is Tuscany. This region registered a high performance in 2007 and was still offering good general assistance in 2012, even improving some hospital and primary care processes (H3, H11, H13, T2).

These two regions (Basilicata and Tuscany) have integrated the IRPES with their governance tools more than the other regions, combining various elements of the ‘hierarchy and targets’ model with elements of ‘transparent public ranking’ and ‘pay for performance’. Information provided by IRPES has been used to set HA targets and define priorities, linking them with the CEO reward management systems. Basilicata and Tuscany publicly disclose their results and disseminate them at the local level through meetings and training programmes for professionals. Marche had a similar performance to Tuscany in 2007, but showed an opposite trend between 2007 and 2012. Its hospital care declined, in terms of appropriateness and quality (H4, H5, H11). This is probably due to a continuous reorganization carried out at the local level and a different approach towards performance evaluation.

A comparison between the autonomous provinces of Trento and Bolzano probably provides the most interesting findings. As Figures 4 and 5 show, the two regions present a similar successful 2007 performance, but with opposite trends, despite similar geographic conditions. They embraced a rather different approach towards performance evaluation: only Trento systematically disclosed and shared data through public meetings, while Bolzano only started in 2014. The different models adopted seem to have affected hospital care appropriateness/efficiency and primary care. Bolzano’s ability to efficiently manage its hospital processes and to divert demand towards the primary care setting seems to have worsened. Trento jointly improved its hospital, primary care and prevention performance. Again, it could be that a combination of ‘hierarchy and targets’/‘transparent public ranking’/‘pay for performance’ governance models are associated with a balanced improvement path.

Umbria and Aosta Valley did not disseminate their IRPES results and they poorly linked the system with other mechanisms, as reported in Section 2. Indeed, their performances have got steadily worse.

Liguria did not use the IRPES in a systematic way, and only slightly improved its 2007 performance.

Finally, the group of regions that adopted a mixed model of governance (group 4) did not benchmark their results against the other regions and only partially disclosed their results. Emilia Romagna, Veneto and Friuli (all of them joined the network after 2012) registered a very high performance in 2007, which declined in 2012 (with the exception of Veneto, which maintained its starting position). This suggests that the mixed model of ‘hierarchy and targets’/‘pay for performance’ alone is not enough to ensure that the high-performing regions keep improving. External benchmarking and public disclosure of data could be a valid incentive to activate peer review processes, reputation pressure and emulate best practices.

## 6 Conclusions

This research draws upon the organizational autonomy Italian regions have been granted since 2001 in order to assess whether different governance models are systematically associated with different performances in the health care sector. None of the regions endorsed a single clear-cut governance model – most combined the five ideal typical models outlined in Section 2. However, an analysis of how they combined these models by using the regional performance management tools in different ways and of the related performance results provides some interesting conclusions.

First, the only region that quite clearly endorsed the ‘choice and competition’ governance model – Lombardy – had a 2012 performance that was above the national average but was nevertheless worse than in 2007. The ‘choice and competition’ governance model by itself does not seem to be associated with a sustained performance improvement.

Second, regardless of the chosen mix of governance models, it could be that external benchmarking represents a precondition to sustained improvement. Rather than exclusively adopting internal benchmarking, a systematic comparison with other providers offers a powerful tool to detect best practices and organizational flaws. It seems that especially high-performing regions – such as Lombardy, Emilia Romagna and Friuli Venezia Giulia – might benefit from comparing themselves with other regions. IRPES can be considered as a starting point in the performance evaluation process, as it provides information that individual regions cannot gather by themselves. Internal benchmarking is important, but it may not be enough to improve regional performance.

Third, despite the fact that no region has exclusively adopted a ‘transparent public ranking’ governance model, our analysis suggests that public disclosure of data can be a powerful tool to drive the improvement in the health care system. This can be explained by the specific lever that public disclosure activates: reputation. This can pave the way to the systematic involvement of clinicians in the improvement process by supporting the identification of best practices and peer review mechanisms.

Fourth, the improvement achieved by two southern regions – Sicily and Basilicata – proves that the coherent adoption of appropriate governance models might help to reduce Italy’s geographical divide.

Further research is needed to understand and analyse if and how the adoption of different governance models affects regional health care performance, by updating available data and examining the impacts of the IRPES on newly adhering regions (Emilia Romagna and Friuli Venezia Giulia).

## Footnotes

1
Legislative Decrees 502/1992 and 517/1993 paved the way for the gradual devolution of health care powers to the regions. The Constitutional Law 3, 18 October 2001 amended articles 114 to 133 of the Italian Constitution and institutionalized regional jurisdiction over the health care sector.

2
Decree Law 56/2000 abolished the national health fund and directly allocated taxes to the regions. A ‘national equalization fund’ was set up to counterbalance differences in regional GDP. Regions are accountable for covering their deficit with their own resources, which include regional taxes and co-payments for health care services.

3
The Financial Stability Law L. 296 (27 December 2006) enforced the pact (Patto per la Salute) that the Italian government and the regional governments had signed on 5 October 2006. The health care funding for the period 2007–2009 was slightly increased and extra funds were allocated to cover 2006 deficits. A specific fund was created for those regions with high deficits, i.e, >7% of the funding (Tediosi *et al.*, 2009; Ferrè *et al.*, 2012).

4
The Lombardy regional Healthcare Directorate, in collaboration with the Interuniversity Research Centre on Public Services, in 2002 started developing a set of performance measurements to systematically evaluate the performance of health care providers in terms of the quality of care. This set of indicators comprises: (1) intra-hospital mortality, (2) mortality within 30 days after discharge, (3) overall mortality (intra-hospital plus within 30 days mortality), (4) voluntary hospital discharges, (5) readmission to an operating room, (6) inter-hospital transfer of patients and (7) readmission for the same major diagnostic categories (Formez, 2007; Vainieri and Nuti, 2011; Vittadini, 2011; Berta *et al.*, 2013).

5
As an example, the timeliness of the surgical treatment for femur-fractured patients can be monitored in different ways, including or excluding specific segments of the population. The highest incidence of fractures refers to people older than 65 and the epidemiological perspective focuses on this cohort, measuring the percentage of femur fractures operated on within two days after the hospital admission among patients older than 65. When used as a performance indicator, however, it overlooks patients younger than 65 and could potentially trigger opportunistic behaviours among clinicians, who might be incentivized to postpone interventions for younger patients in order to achieve the target. By endorsing a ‘managerial’ perspective, IRPES chose to include all the patients in the computation of the indicator, in order to prevent these kinds of side effects.

6
The replication data set can be accessed via the electronic version of *Health Economics*, *Policy and Law*.
